# Deep learning-enhanced microscopy with extended depth-of-field

**DOI:** 10.1038/s41377-023-01323-y

**Published:** 2023-11-24

**Authors:** Yide Zhang

**Affiliations:** Caltech Optical Imaging Laboratory, Andrew and Peggy Cherng Department of Medical Engineering, Department of Electrical Engineering, California Institute of Technology, Pasadena, CA 91125 USA

**Keywords:** Wide-field fluorescence microscopy, Imaging and sensing

## Abstract

A computational imaging platform utilizing a physics-incorporated, deep-learned design of binary phase filter and a jointly optimized deconvolution neural network has been reported, achieving high-resolution, high-contrast imaging over extended depth ranges without the need for serial refocusing.

Optical microscopy has served as an indispensable tool across a multitude of scientific disciplines and clinical applications. Despite its transformative impact, traditional optical systems are constrained by intrinsic limitations such as a restricted depth-of-field (DoF). The DoF is a critical parameter that defines the depth range of an object that can be sharply imaged by a given optical imaging system. It is defined as

$${\rm{DoF}}=\frac{n\cdot \lambda }{{{\rm{NA}}}^{2}}+\frac{n}{M\cdot {\rm{NA}}}e,$$

where *n* is the refractive index of the imaging medium, *λ* denotes the wavelength of the light, NA is the numerical aperture of the objective, *M* is the magnification factor of the microscope, and *e* represents the pixel pitch of the image sensor. According to the definition, extending the DoF is feasible by reducing the NA. However, the NA also dictates the system’s spatial resolution, as governed by the Abbe diffraction limit, defined as *λ*/2NA. Therefore, an intrinsic trade-off exists between extending the DoF and maintaining high spatial resolution. To accomplish both, conventional approaches often require a tedious process of iterative refocusing for different axial positions of the sample. This compromise poses substantial challenges in applications like cytometry^1^, histology^2^, and endoscopy^3^, where the imperative for high-resolution imaging over an expansive axial range is critical.

To address this issue, recent attention has shifted towards binary phase filters (BPFs)^4–6^. These filters, composed of concentric rings with alternating phases, offer a promising avenue for extending the DoF without degrading the spatial resolution of the microscope. What makes BPFs particularly appealing is their simplicity and ease of manufacturing. Researchers have been drawn to explore their potential to overcome the DoF limitations in conventional optical microscopy. However, designing BPFs with more than five concentric rings has proven to be a daunting computational task, characterized by intricate non-linear equations^7^. This computational complexity has, in the past, acted as a bottleneck, limiting researchers from exploring the full potential of BPFs in overcoming the DoF limitations in optical microscopy.

In a recently published paper in *Light: Science & Applications*, a team of researchers from Yonsei University, Seoul, Republic of Korea, have proposed an innovative solution to these challenges^8^. Their approach termed the E2E-BPF microscope—an acronym for “end-to-end optimized binary phase filter”—redefines the possibilities of optical microscopy by enabling high-resolution imaging across an extended DoF without the need for serial refocusing. E2E-BPF seamlessly blends principles of physics with the power of deep learning techniques^9^. The key innovation in E2E-BPF is the combination of physics-based principles with deep learning techniques, leading to the design of a BPF that can be effectively tailored to extend the DoF (Fig. 1a). Diverging from traditional approaches that face limitations related to the number of concentric rings in the BPF, the E2E-BPF microscope leverages a deep learning framework that jointly optimizes the BPF design and the relevant U-net-based imaging reconstruction network using a vast dataset^10^ (Fig. 1b). This synergistic approach allows the design of BPFs with a larger number of concentric rings, thereby dramatically extending the DoF by a factor of 15.5 (using 64 design variables) when compared to conventional optical systems.

**Fig. 1:**
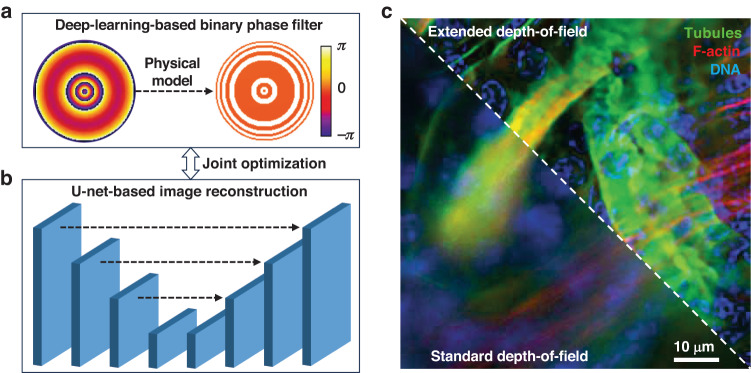
Illustration of the concept of E2E-BPF microscopy reported in ref. ^8^ **a** Optimization of the deep-learned design of the binary phase filter, which is initialized with a continuous axisymmetric function, based on the physical model describing the imaging process. **b** U-net-based image reconstruction network that is jointly optimized with the binary phase filter in (**a**). **c** Comparison of the multicolor fluorescent images of a mouse kidney tissue section (tubules stained with AF488, F-actin filaments stained with AF568, DNA stained with DAPI) acquired with the standard (bottom-left) and E2E-BPF (top-right) microscopes, which have standard and extended depths-of-field, respectively

The efficacy of the E2E-BPF microscope was evaluated through numerical simulations and experimental validation. The authors employed both fluorescently labeled beads and histological sections of biological tissues as test samples. Notably, they demonstrated its biological viability by imaging cellular specimens and a thick mouse kidney tissue section stained with fluorescent dyes—all achieved without the need for refocusing (Fig. 1c). The results consistently indicated a substantial enhancement in resolution and contrast while extending the DoF significantly. In addition, the time required for image acquisition and processing was reduced by a factor of 15.5, substantially alleviating the time burden typically associated with conventional imaging techniques requiring serial refocusing.

The potential applications of the E2E-BPF microscope are broad and far-reaching. It offers a highly effective and scalable strategy for DoF extension in optical imaging systems. One particularly notable application is in light sheet fluorescence microscopy, where extended DoF and high light efficiency are critical^6^. The E2E-BPF can be tailored to generate sharp and elongated excitation light sheets, enhancing the visualization of fluorescent samples in three dimensions. This opens new possibilities for high-throughput, high-resolution volumetric imaging without the need for serial refocusing. Furthermore, the E2E-BPF platform has the potential to benefit other 3D imaging modalities, such as optical coherence tomography^11^ and photoacoustic microscopy^12^. These techniques also demand high-resolution imaging over an extended DoF, and the E2E-BPF promises to enhance their imaging performance and broaden their applications.

The impact of this work is significant and multifaceted. It is poised to revolutionize rapid image-based diagnosis, optical vision, and metrology by providing a tool that overcomes the limitations of conventional microscopy. Looking ahead, there are several exciting directions for future research. Incorporating system aberrations into the design framework presents an opportunity to further enhance image quality. This strategy can be extended to handle spatially varying aberrations in 3D microscopes. Additionally, addressing aberrations stemming from mismatches between immersion liquids and imaging samples, a common issue in high-NA imaging systems, is a promising avenue. Importantly, the E2E-BPF platform exhibits the potential for even greater DoF extension. The authors have successfully designed BPFs that achieve an even greater 22.08×-DoF extension using 128 design variables. This underscores the versatility and scalability of the E2E-BPF approach, hinting at its continued impact on the field of optical imaging.

## References

[1] Meng Q (2022). A drop-in, focus-extending phase mask simplifies microscopic and microfluidic imaging systems for cost-effective point-of-care diagnostics. Anal. Chem..

[2] Jin LB (2020). Deep learning extended depth-of-field microscope for fast and slide-free histology. Proc. Natl Acad. Sci. USA.

[3] Kim J (2017). Endoscopic micro-optical coherence tomography with extended depth of focus using a binary phase spatial filter. Opt. Lett..

[4] Martínez-Corral M (1995). Tunable axial superresolution by annular binary filters. Application to confocal microscopy. Opt. Commun..

[5] Liu LB (2007). Binary-phase spatial filter for real-time swept-source optical coherence microscopy. Opt. Lett..

[6] Ryu S (2020). Light sheet fluorescence microscopy using axi-symmetric binary phase filters. Biomed. Opt. Express.

[7] Falcón R (2017). Performance limits of binary annular phase masks codesigned for depth-of-field extension. Opt. Eng..

[8] Seong B (2023). E2E-BPF microscope: extended depth-of-field microscopy using learning-based implementation of binary phase filter and image deconvolution. Light Sci. Appl.

[9] Karniadakis GE (2021). Physics-informed machine learning. Nat. Rev. Phys..

[10] Ronneberger, O., Fischer, P. & Brox, T. U-Net: convolutional networks for biomedical image segmentation. In *Medical Image Computing and Computer-Assisted Intervention – MICCAI 2015*. MICCAI 2015, Vol 9351 (eds Navab, N., Hornegger, J., Wells, W., Frangi, A.) Lecture Notes in Computer Science, (Springer, Cham, 2015).

[11] Huang D (1991). Optical coherence tomography. Science.

[12] Cao R (2023). Optical-resolution photoacoustic microscopy with a needle-shaped beam. Nat. Photonics.

